# Prevalence of Malnutrition and Associated Factors in Older Patients with Rheumatoid Arthritis: A Cross-Sectional Study

**DOI:** 10.3390/nu15163500

**Published:** 2023-08-08

**Authors:** Laura Cano-García, Rocío Redondo-Rodríguez, Sara Manrique-Arija, Carmen Domínguez-Quesada, Juan Crisóstomo Vacas, Pedro Armenteros-Ortiz, Desiree Ruiz-Vilchez, José María Martín-Martín, Aimara García-Studer, Fernando Ortiz-Márquez, Natalia Mena-Vázquez, Antonio Fernández-Nebro

**Affiliations:** 1 Instituto de Investigación Biomédica de Málaga (IBIMA)-Plataforma Bionand, 29010 Málaga, Spain; lauracano.due@gmail.com (L.C.-G.); rocioredondo91@hotmail.com (R.R.-R.); sarama_82@hotmail.com (S.M.-A.); aimara.garcia95@gmail.com (A.G.-S.); fomquez@gmail.com (F.O.-M.); afnebro@gmail.com (A.F.-N.); 2 UGC de Reumatología, Hospital Regional Universitario de Málaga, 29009 Málaga, Spain; 3 Departamento de Medicina, Universidad de Málaga, 29010 Málaga, Spain; 4 UGC de Reumatología, Hospital Universitario Virgen Macarena, 41701 Sevilla, Spain; cdq.due@gmail.com; 5 UGC de Reumatología, Hospital Universitario Reina Sofía de Córdoba, 14004 Córdoba, Spain; juancris61@hotmail.com (J.C.V.); pedroj.armemteros.sspa@juntadeandalucia.es (P.A.-O.); desiree.ruiz@hotmail.com (D.R.-V.); 6 Instituto Maimónides de Investigación Biomédica de Córdoba (IMIBIC), 14004 Córdoba, Spain; 7 UGC de Reumatología, Hospital Universitario Nuestra Señora de la Candelaria, 38010 Santa Cruz de Tenerife, Spain; chema904@hotmail.com

**Keywords:** rheumatoid arthritis, elderly, malnutrition, inflammation, quality of life, physical function

## Abstract

Objective: To describe the frequency of malnutrition in older patients with rheumatoid arthritis (RA) and investigate associated risk factors. Methods: This multicenter, cross-sectional study included participants aged ≥65 years who met the 2010 ACR/EULAR criteria for RA. Nutritional status was assessed using the Mini Nutritional Assessment Short Form (MNA-SF) and based on variables, such as albumin level, the Geriatric Nutritional Risk Index (GNRI), and vitamin D. Data were also collected on epidemiological variables, inflammatory disease activity, quality of life, physical function, and frailty. Multivariate models were used to study factors associated with nutritional status. Results: The study population comprised 76 RA patients aged ≥65 years, of whom 68.4% had a normal nutritional status, and 31.5% had an impaired nutritional status: 28.9% were at risk of malnutrition, and 2.6% were malnourished. Additionally, 10% had albumin levels <3.8 g/L. Patients with impaired nutritional status had poorer quality of life and physical function. The factors associated with compromised nutritional status (OR [95% CI]) were age (1.0 [1.0–1.1]; *p* = 0.035), DAS28-ESR (1.8 [1.0–3.2]; *p* = 0.024), and EuroQoL-5D-5L (0.9 [0.9–0.9]; *p* = 0.040). Furthermore, the GNRI was associated with the MNA score (0.06 [0.0–0.1]; *p* = 0.014). Conclusions: Approximately one-third of older patients with RA have impaired nutritional status. Older age, higher inflammatory disease activity, and decreased quality of life are associated with impaired nutritional status. The MNA and GNRI are valuable tools for assessing the nutritional status of patients with RA.

## 1. Introduction

Rheumatoid arthritis (RA) is a chronic immune-mediated inflammatory disease of unknown etiology. When insufficiently treated, it severely affects the joints and is associated with disability; multimorbidity; reduced life expectancy; and increased health, social, and economic burden. While onset can be at any stage of life, its prevalence increases with age. Recent studies suggest that older patients experience more severe RA, with poorer health outcomes [1,2]. Furthermore, given their poorer health, older patients with RA may receive less intensive treatment, with reduced prescription of biologics and conventional synthetic disease-modifying antirheumatic drugs (csDMARDs), thus potentially leading to a more severe impact on their health [3].

Patients with RA, especially older patients, are prone to debilitating extra-articular manifestations, such as loss of appetite, sarcopenia, fever, weakness, fatigue, and neuropsychiatric disorders [4]. An association has been identified between body weight and inflammatory disease activity, especially in RA. Some studies have reported a hypercatabolic–anorexic state known as “rheumatoid cachexia”, which is associated with inflammation itself, mainly affecting muscle mass [5,6]. Similarly, obesity has been associated with inflammation, for the most part via the increased production of proinflammatory lipokines and cytokines in adipose tissue [7]. In any case, both sarcopenic obesity, which is characterized by reduced lean mass with adiposity, and malnutrition have been associated with increased inflammatory disease activity and poorer outcomes in RA [8]. 

The prevalence of malnutrition and its impact on mortality and quality of life have been evaluated in many chronic diseases. A negative impact on quality of life has been reported for patients with cardiovascular diseases [9], respiratory diseases [10], and cancer [11]. However, this aspect has received little attention in RA [4,12,13]. One study found that malnourished patients with RA had poorer functional prognoses and reduced life expectancy [13]. Another study found that affected patients had poorer quality of life and were more frail than well-nourished persons [4]. Nevertheless, little is known about the association between malnutrition and other clinical and laboratory variables and physical function, even though the variables could have a notable impact on disease progression. Analyzing these aspects will provide valuable information that enables us to better understand the effects of malnutrition on patients with RA, in addition to their influence on various aspects of health and well-being. Moreover, it is possible to develop more effective interventional strategies to improve patients’ quality of life and identify possible risk factors and clinical markers that can help to detect and manage malnutrition in the early stages. Consequently, the objective of the present study was to describe malnutrition in older patients with RA and to evaluate its association with various clinical factors, such as quality of life, physical function, frailty, morbidity, and polymedication. 

## 2. Materials and Methods

### 2.1. Design and Data Source

We performed a multicenter, cross-sectional descriptive study. Data were collected at 4 Spanish university hospitals: Hospital Universitario Regional de Málaga, Hospital Universitario Virgen Macarena Sevilla, Hospital Universitario Reina Sofía de Córdoba, and Hospital Universitario Nuestra Señora de la Candelaria. The information was recorded at specialist clinics with rheumatologists and nurses who were fully trained in the management of RA. Before inclusion, all participants gave their written informed consent. Our research fulfilled the ethics criteria of the Declaration of Helsinki, and the study protocol was approved by the Ethics Committee of Málaga (code no.: 2406-N-20).

### 2.2. Eligibility

We consecutively recruited all participants between April and December 2021. The inclusion criteria were those of the 2010 ACR/EULAR classification of RA [14] and comprised onset of disease at age > 16 years, age ≥ 65 years at inclusion, and ability to complete the questionnaire. We excluded patients with the following: inflammatory diseases other than RA (except for secondary Sjögren syndrome), active infection, and dementia or cognitive impairment that made it difficult to provide accurate answers.

### 2.3. Study Protocol

At the study centers, patients with RA receive care jointly from a rheumatologist and a specialist nurse. They are usually seen every 3–6 months or more frequently, if necessary, from a clinical perspective. Patients were invited to participate in the study by the reference rheumatologist. Once they had given their written informed consent, the selection criteria were verified, and clinical data were recorded according to the data collection protocol. The questionnaires were completed at the nursing clinic, and anthropometric data were recorded. Biological samples were taken after a minimum 8 h fast.

### 2.4. Main Outcome Measure and Covariates

#### 2.4.1. Nutritional Status

The main outcome measure of the study was nutritional status. This was evaluated using the Mini Nutritional Assessment Short Form (MNA-SF) [15,16], which is a short questionnaire applied to assess the nutritional status of a single individual. It consists of 6 questions and focuses on aspects associated with food intake, weight loss, mobility, psychological stress, and acute/chronic diseases. The responses are used to calculate a total score, which ranges from 0 (poorest nutritional status) to 14 (optimal nutritional status). According to the score obtained, the participants’ nutritional status was classified as follows: (1) normal (>11 points), (2) risk of malnutrition (between 8 and 11 points), and (3) malnutrition (between 0 and 7 points). Patients were stratified for analysis into two groups: normal nutritional status (category 1) and impaired nutritional status (categories 2 and 3). 

Nutritional status was also evaluated based on plasma levels of vitamins D and B12; total proteins; albumin; and the Geriatric Nutritional Risk Index (GNRI), which is calculated using blood albumin levels, actual body weight, and ideal weight (kg), according to the following formula: GNRI = (1.489 × Alb [g/L]) + (41.7 × [actual weight/ideal weight]). The result is a number that usually ranges from 0 to 130; a higher GNRI value indicates a lower risk of malnutrition. The index can be classified as follows: (1) normal (>98), (2) low risk (between 98 and 92), (3) moderate risk (between 92 and 82), and (4) high risk of malnutrition (<82) [17].

#### 2.4.2. Epidemiological Variables

We collected epidemiological data from all the participants, including age, sex, educational level, and income. We recorded general comorbid conditions using the Charlson index, which takes into account 19 predefined clinical conditions with differently weighted values. We also applied the age-adjusted Charlson index, which adapts the Charlson index according to specific age ranges [18,19]. In addition, we considered traditional cardiovascular risk factors, such as smoking, obesity, arterial hypertension, diabetes mellitus, dyslipidemia, and sedentary lifestyle [20].

#### 2.4.3. Quality of Life, Physical Function, and Frailty 

We used the 5-dimension 5-level EuroQoL questionnaire (EQ-5D-5L) to evaluate health-related quality of life. The questionnaire consists of a series of questions for the evaluation of 5 dimensions: mobility, self-care, usual activities, pain/discomfort, and anxiety/depression. It also includes a visual analog scale (VAS) known as EQ-VAS. Lower values on the scale and questionnaire represent poorer health status, whereas higher values indicate better health status [21,22]. 

Physical function was assessed using 3 instruments: the International Physical Activity Questionnaire (IPAQ) [23], the Health Assessment Questionnaire (HAQ) [24], and the Steinbrocker functional classification [25]. The IPAQ is based on self-reported physical activity and collects information on the frequency and duration of various activities [23]. The HAQ comprises a series of questions addressing 8 areas of daily life, including dressing and grooming, arising, eating, walking, and domestic tasks. The value assigned to each question ranges from 0 (no involvement) to 3 (maximum involvement) [24]. Lastly, the Steinbrocker classification is used in RA to describe the degree of joint involvement and functional impairment. It is divided into different stages, ranging from stage I (performing all usual activities without limitation) to stage IV (incapacitated, largely or wholly bedridden, or confined to a wheelchair with little or no self-care) [25]. 

The Short Physical Performance Battery (SPPB) is used to evaluate physical function and frailty in older adults using 3 main areas: walking, sit-to-stand, and balance. The SPPB score ranges from 0 to 12 points, where a higher score is indicative of better physical function. According to the EWGSOP2 guidelines [26], a score under 10 on the SPPB is indicative of impaired physical performance [8].

#### 2.4.4. Other Variables Associated with RA

During the inclusion phase, we collected multiple RA-related variables, including duration of symptoms, disease activity according to the 28-joint Disease Activity Score with erythrocyte sedimentation rate (DAS28-ESR) [27] and C-reactive protein (CRP) levels (mg/L). CRP levels < 5 mg/L were considered low, and those ≥5 mg/L were considered high [28,29]. We also collected data associated with disease severity, such as rheumatoid factor (U/mL) and anti-citrullinated peptide antibodies (ACPA) (U/mL), with values above 20 and 10, respectively, considered positive. Furthermore, we recorded the presence of radiological erosions, when at least 1 erosion on hands/feet was detected. As for treatment, we collected data on the use of DMARDs (including csDMARDs and biological DMARDs (bDMARDs)) and corticosteroids at inclusion.

### 2.5. Statistical Analysis

We performed a descriptive analysis of the data using absolute frequencies and percentages or mean and standard deviation (SD) or median and interquartile range (IQR). We verified the normality of the distribution using the Kolmogorov–Smirnov test. Patients with normal and impaired nutritional status were compared using the Pearson χ^2^ test for qualitative variables and the *t* test for quantitative variables, as applicable. 

We ran two multivariate models, one based on logistic regression to investigate the variables that were independently associated with impaired nutritional status (MNA ≤ 11 points) and another based on linear regression for the dependent variable MNA (0–14 points). The model included all the variables that proved to be significant in the bivariate analysis and those that were of clinical interest. Given an alpha risk of 0.10 and a beta risk of 0.2 in a bilateral contrast, the sample size calculation showed that 72 patients were necessary to detect an expected significant difference in perceived QoL, where patients with a normal body weight scored higher than those who were at risk of malnutrition [4]. Statistical significance was set at *p* < 0.05. All the statistical analyses were performed using IBM SPSS Statistics for Windows, Version 28 (IBM Corp., Armonk, NY, USA).

## 3. Results

### 3.1. Baseline Characteristics

We recruited 76 RA patients aged ≥65 years. Table 1 shows the main baseline characteristics. Most patients were women (78.9%), and the mean (SD) age was 71 (4.8) years. Almost three-quarters were educated to primary level (72%), and two-thirds earned less than EUR 1500 per month (67%). Associated disease was recorded in 82% of patients, with a median (IQR) age-CCI of 3.0 (2.0–3.0). Arterial hypertension was the most common comorbid condition (56%), followed by dyslipidemia (39%) and osteoporosis (19%). The mean body mass index was 28.1, indicating moderate overweight. None of the patients in our sample had comorbid gastrointestinal conditions, including inflammatory bowel disease, peptic ulcer, or intestinal surgery. 

As for clinical characteristics, the mean time since diagnosis was 18 years, and most patients had seropositive disease (75%) and erosive disease (85%). Mean inflammatory disease activity was 2.9 according to the DAS28-ESR, and 64.5% of patients were in remission or had low disease activity, whereas 35.5% had moderate or high activity. Similarly, 36% of patients had high CRP values (≥5 mg/L). More than half of the patients were taking a csDMARD (60%) and a bDMARD (73%). 

### 3.2. Nutrition, Physical Function, and Quality of Life

As seen in Table 2, patients with RA had a mean (SD) MNA index of 12.3 (2.0). We can also see that 52/76 (68.4%) were within the normal range, whereas 24/76 (31.5%) had impaired nutritional status: 28.9% were at risk of malnutrition, and 2.6% were malnourished. While the mean levels of total proteins and albumin were normal, albumin levels were <3.8 g/L in almost 10% of patients, and the GNRI was low or moderate in most.

Quality of life was moderately affected, with a median EQ-5D-5L score of 0.53 and an EQ-VAS score of 55.0. The HAQ yielded a mean score of 1.282, indicating a mild-to-moderate limitation in activities of daily living. According to the Steinbocker functional classification, most patients were fully independent for activities of daily living (Table 2).

### 3.3. Factors Associated with Malnutrition

Table 3 shows the characteristics of RA patients aged ≥65 years according to nutritional status. No significant differences were found for sex. However, differences were observed with respect to age since the mean age of patients with impaired nutritional status was higher than in the group with normal nutritional status (*p* = 0.007). By age group, patients with impaired nutrition were aged 80 to 90 years, and those with optimal nutrition were aged 65 to 69 years. As for disease characteristics, significantly higher values were recorded for RA patients with impaired nutritional status in disease duration (*p* = 0.042) and inflammatory disease activity according to the DAS28-ESR (*p* = 0.003), SDAI (*p* = 0.010), CDAI (*p* = 0.013), and CRP ≥ 5 g/L (*p* = 0.033). No significant differences were found between the two groups for pharmacological treatment.

The comparison of other nutritional parameters between the groups revealed that patients with an impaired nutritional status according to the MNA also had a greater risk of severe malnutrition according to the GNRI (16.7% vs. 1.9%; *p* = 0.034) and more frequently had albumin levels < 3.8 g/L (20.8% vs. 3.8%; *p* = 0.017). Moreover, significant differences were found in vitamin D levels, for which levels in the normal nutrition group were higher than in the impaired nutrition group (*p* = 0.035).

Finally, we found that patients with RA and impaired nutritional status had poorer quality of life measured using the EQ-5D-5L (*p* = 0.001), lower levels of physical activity in METs (*p* = 0.014), and poorer physical function according to the HAQ (*p* = 0.044) and the SPPB questionnaire (*p* = 0.025).

**Figure 1:**
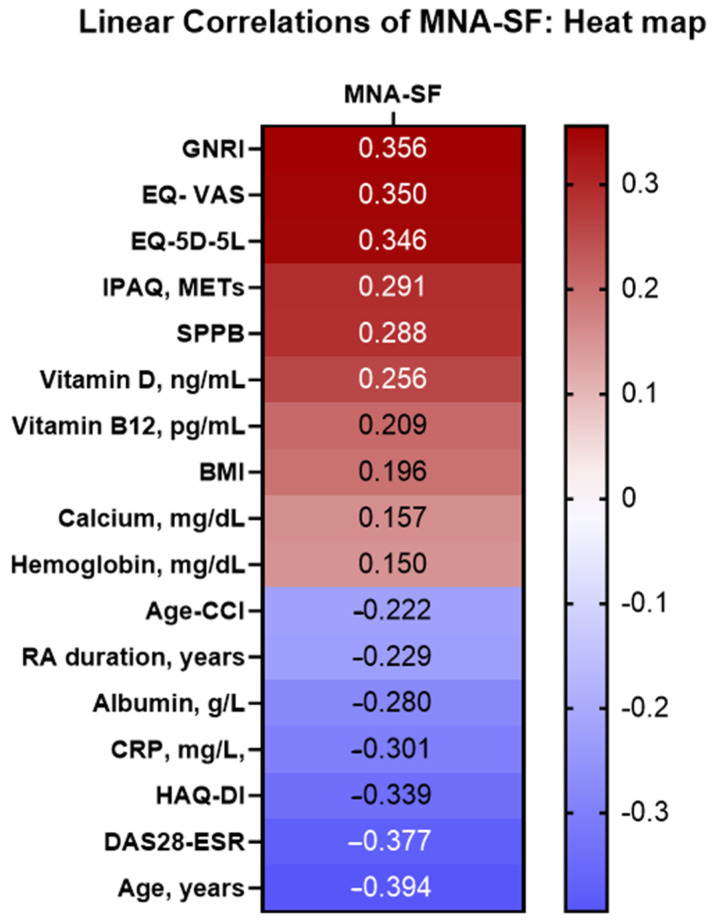
Analysis of the linear correlation between MNA and the characteristics of 76 older patients (≥65 years) with RA. Abbreviations: RA: Rheumatoid arthritis; MNA: MNA: Mini Nutritional Assessment; Age-CCI: age-adjusted Charlson Comorbidity Index; BMI: body mass index; DAS28-ESR: 28-joint Disease Activity Score with erythrocyte sedimentation rate; SDAI: Simple Disease Activity Index; CDAI: Clinical Disease Activity Index; CRP: C-reactive protein; GNRI: Geriatric Nutritional Risk Index; EQ-5D-5L: 5-dimension 5-level European Quality of Life questionnaire; VAS: visual analog scale; IPAQ: International Physical Activity Questionnaire; HAQ-DI: Health Assessment Questionnaire Disability Index; SPPB: Short Physical Performance Battery.

**Figure 2:**
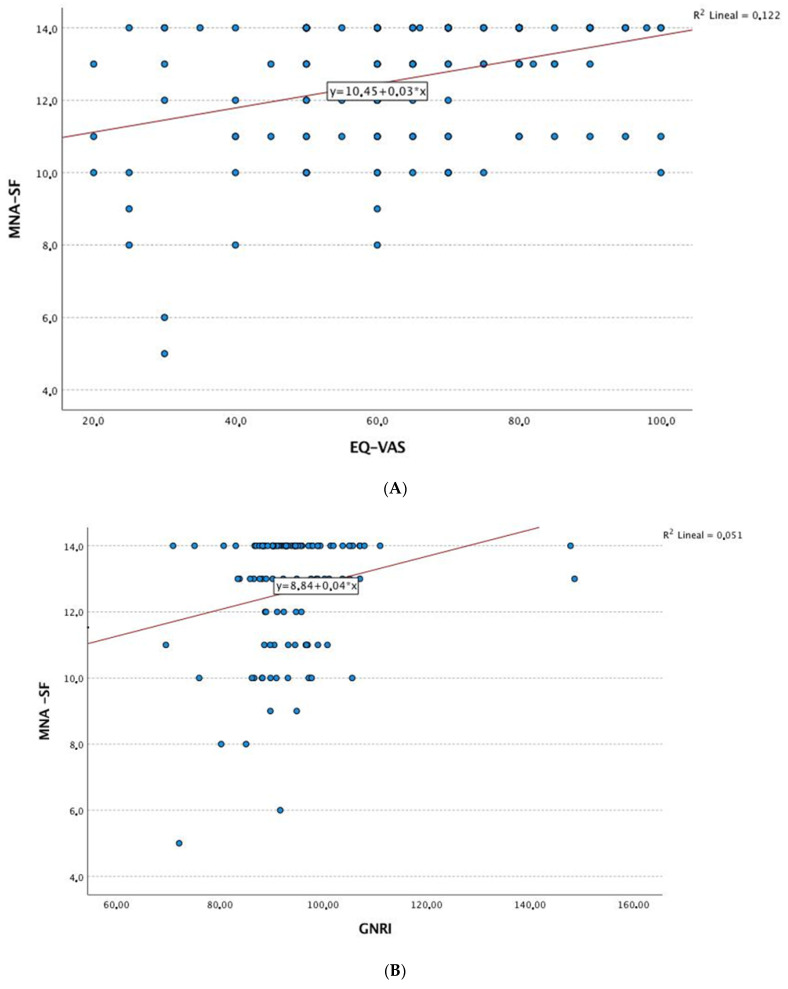

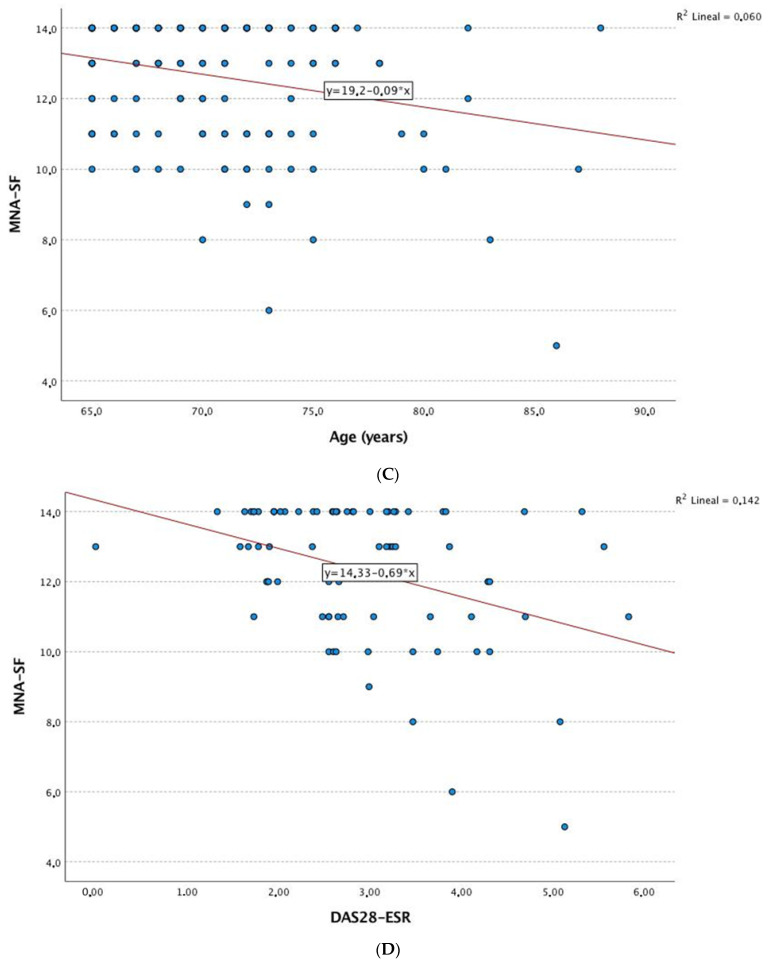
Correlation analysis. (**A**) Linear correlation analysis between MNA and EQ-VAS. (**B**) Linear correlation analysis between MNA and GNRI. (**C**) Linear correlation analysis between MNA and age (years). (**D**) Linear correlation analysis between MNA and DAS-ESR. Abbreviations: MNA: Mini Nutritional Assessment; GNRI: Geriatric Nutritional Risk Index; EQ-VAS: 5-level 5-dimension European Quality of Life questionnaire visual analog scale; DAS28-ESR: 28-joint Disease Activity Score with erythrocyte sedimentation rate.

Figure 1 shows the correlations between nutritional status by the MNA and a series of clinical and quality-of-life variables in patients with RA. The results showed that age, disease duration, inflammatory disease activity, and laboratory parameters (e.g., albumin, hemoglobin, and vitamin D) were negatively correlated with the MNA score. Furthermore, positive correlations were detected between the MNA and quality of life, physical activity, and the SPPB result (Figure 2).

### 3.4. Multivariate Analysis

Table 4 shows the results of the multivariate logistic regression analysis for the dependent variable impaired nutritional status according to the MNA score in patients with RA. Age and inflammatory disease activity (DAS28-ESR) were independently associated with impaired nutritional status, whereas better physical function (EQ-VAS) was a protective factor. 

**Figure 3:**
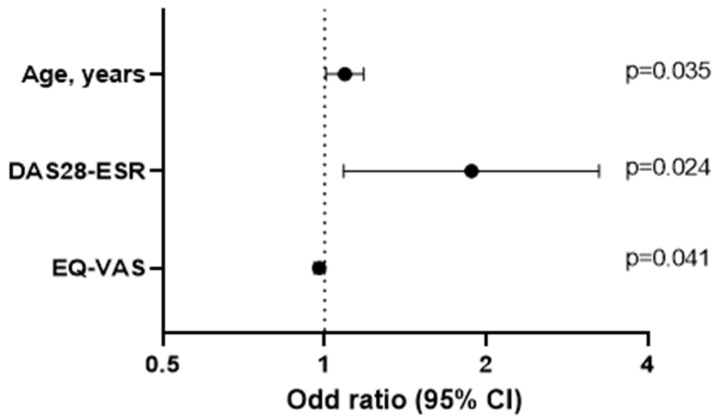
Logistic regression analysis.

Similarly, Table 5 shows an alternative multivariate analysis based on linear regression, with the MNA as the dependent variable (0 to 14 points, with 0 representing the worst nutritional status and 14 representing the best). In this model, the MNA score remained associated with age (β = −0.294); inflammatory disease activity was evaluated using the DAS28-ESR (β = −0.236); and quality of life was evaluated using the EQ-VAS (β = 0.229) and the GNRI value (β = 0.251) (Figure 3).

## 4. Discussion

Malnutrition has a significant impact on the quality of life of older patients with chronic diseases [30]. However, little research has been performed on the problems caused by malnutrition in older patients with RA and associated factors. The present study addressed this shortcoming and showed that nutritional status was impaired in approximately one-third of older patients with RA. Interestingly, nutrition was deficient in 29% of patients, who will be at risk of malnutrition in the future if they do not take appropriate measures, whereas in 3%, malnutrition was sufficiently serious to warrant immediate intervention. Furthermore, albumin levels were <3.8 g/L in around 10% of patients, pointing to possible nutritional dysfunction. These data highlight the importance of evaluating and addressing nutritional status in older patients with RA in order to prevent negative impacts on their health and general well-being and potential interference with the treatment of RA. 

Previous studies on the prevalence of malnutrition in RA have used different measures to assess nutritional status and cachexia, including body mass index, triceps skinfold thickness, arm muscle area, and biochemical parameters (albumin and cholesterol levels). Based on these parameters, the prevalence of malnutrition in RA ranges from 20% to 50% [5,31,32,33]. Nevertheless, specific questionnaires, such as the MNA, have been designed for the rapid and accurate evaluation of malnutrition. However, the MNA is not designed to detect specific nutritional deficiencies and should be complemented by other instruments. Tański et al. [4] found that approximately 42% of older patients with RA have impaired nutritional status according to the MNA, with 6% classified as malnourished. These results show a slightly higher prevalence of malnutrition than in our study, possibly because the patients assessed had a higher mean age and more frequent gastrointestinal involvement.

As for factors associated with impaired nutritional status in RA, we found that clinical factors such as age and inflammatory disease activity played a role. Several studies that have evaluated malnutrition in persons aged ≥65 years stress that the risk of malnutrition increases with age and the level of medical care required [34,35,36]. As we get older, our body undergoes physiological changes that affect the nervous, endocrine, gastrointestinal, renal, and muscle systems, which can in turn affect appetite, the absorption of nutrients, and metabolism. This can lead to reduced food intake and less efficiency in the use of nutrients. The resulting effects can worsen in patients with chronic inflammatory diseases, for example, RA, which is affected by other factors such as the use of medication and inflammation [34].

Furthermore, several studies have shown a relationship between inflammatory disease activity, cachexia, and sarcopenic obesity in RA. High levels of inflammatory cytokines, such as tumor necrosis factor α (TNF-α), interleukin (IL) 1β, IL-6, and CRP, together with loss of muscle mass, could account for malnutrition in affected patients [4,37]. We found an association between impaired nutritional status according to the MNA and all the measures of inflammatory disease activity, especially DAS28-ESR and elevated CRP levels. 

These findings suggest that disease is more severe in patients with impaired nutritional status and that, therefore, systemic inflammation and inflammatory mediators can affect nutrient metabolism, cause loss of muscle mass, and affect food intake, potentially contributing to malnutrition. In line with this observation, Tokumoto et al. [38] found an association between DAS28-ESR and malnutrition according to the GNRI. The interrelationships between all these factors have been recently reviewed [39]. According to this review, a balanced and anti-inflammatory diet is essential for individuals with rheumatoid arthritis and sarcopenia. Including foods rich in omega-3 fatty acids (found in fatty fish, chia seeds, flaxseeds, etc.), antioxidants (found in colorful fruits and vegetables), and lean proteins can help support muscle health and reduce inflammation. These types of dietary interventions can complement other interventions like exercise and potentially improve muscle health in individuals with rheumatoid arthritis and sarcopenia. However, more research is needed to establish specific dietary guidelines tailored to this population. However, Markaki et al. [37] suggested that the patient-generated subjective global assessment questionnaire may not have been sufficiently sensitive to represent the association between malnutrition and inflammatory disease activity in patients with RA.

Similarly, older patients with RA and impaired nutritional status were characterized by poorer quality of life (EQ-VAS), lower levels of physical activity (METs), and deficient physical function (HAQ and SPPB). The multivariate analysis showed that EQ-VAS was independently associated with impaired nutritional status. These findings are consistent with those of other studies showing that patients with RA and malnutrition experience more pronounced impairment in their quality of life than those with normal nutritional status, even in the context of RA in older patients [4,40,41]. Fukuda et al. [40] found that loss of muscle protein is a crucial factor in the impairment of quality of life, independently of disease activity. Therefore, it is essential that the treatment of RA includes appropriate nutritional management. 

Our study showed that the GNRI was significantly associated with the MNA. This finding highlights the role of albumin deficiency in impaired nutritional status in patients with RA. As the GNRI does not involve interviews with patients, it does not collect information on dietary habits and daily life, although it does evaluate nutritional risk based on serum albumin and body weight [42]. While we were unable to find studies that specifically address the association between the MNA and GNRI in patients with RA, studies performed in healthy older individuals have shown that the validity of the GNRI could be compared with that of the MNA. Thus, it has been suggested that the GNRI could complement the information on nutrition provided by the MNA [43]. 

Our study has both limitations and strengths. First, our sample size was relatively small, and the design was cross-sectional. However, the sample size was adjusted according to the sample size calculation and proved sufficient to describe the frequency of malnutrition and associated factors in older patients with RA. Similarly, restricting the study population to patients aged ≥65 years prevents our results from being generalized to younger populations. Of note, we evaluated malnutrition using the MNA questionnaire, which is completed based on the patient’s subjective opinion. Nevertheless, the MNA is widely used and has been validated in geriatrics to identify older persons at risk of malnutrition or who are malnourished [44]. Furthermore, we complemented the information obtained using the MNA with other nutritional parameters, such as the GNRI, which considers albumin values and body weight. Finally, we have not specifically studied the gastrointestinal manifestations of RA patients; however, we took into account some digestive comorbidities that can influence nutrition such as inflammatory bowel disease, peptic ulcer, or intestinal surgery.

## 5. Conclusions

Our results showed that nutritional status is impaired in around one-third of RA patients aged ≥65 years, who either are malnourished or are at risk of malnutrition. Older age, higher inflammatory disease activity, and decreased quality of life are independently associated with impaired nutritional status in RA patients aged ≥65 years. The MNA, together with albumin and vitamin D levels, could be routinely used to detect the risk of malnutrition and apply nutritional intervention strategies that improve health outcomes in older patients with RA. Controlled clinical trials are necessary to evaluate the impact of nutritional interventions on quality of life, physical function, inflammation, and even mortality in older patients with RA.

## Figures and Tables

**Table 1:** Baseline characteristics of 76 older patients (≥65 years) with RA.

Variable	RA ≥ 65 Years = 76
Epidemiologic characteristics	
Female sex, n (%)	60 (78.9)
Age in years, mean (SD)	71.0 (4.8)
Educational level	
No studies, n (%)	9 (11.8)
Primary studies, n (%)	55 (72.4)
Secondary studies, n (%)	10 (13.2)
Higher education, n (%)	2 (2.6)
Economic level	
No income, n (%)	12 (15.8)
Income < EUR 1500, n (%)	51 (67.1)
Income ≥ EUR 1500, n (%)	13 (17.1)
Clinical characteristics	
Comorbid conditions	
High blood pressure, n (%)	43 (56.6)
Diabetes mellitus, n (%)	13 (17.1)
Dyslipidemia, n (%)	30 (39.5)
Cardiovascular disease, n (%)	4 (5.3)
Osteoporosis, n (%)	15 (19.7)
Asthma, n (%)	1 (7.9)
Other comorbidities, n (%)	44 (57.9)
Smoking	
Non-smokers, n (%)	54 (71)
Smokers, n (%)	6 (7.9)
Former smokers, n (%)	16 (21.1)
Age-CCI, median (IQR)	3.0 (3.0–4.0)
Body mass index, mean (SD)	28.1 (5.0)
Time since diagnosis of RA in years, mean (SD)	18 (7.8)
Positive rheumatoid factor (>10), n (%)	57 (75.0)
Positive anti-citrullinated peptide antibodies (>20), n (%)	55 (72.4)
Erosive disease, n (%)	65 (85.5)
Inflammatory disease activity	
DAS28-ESR at protocol, score 0–10, mean (SD)	2.9 (1.1)
Remission or low activity, n (%)	49 (64.5)
Moderate or high activity, n (%)	27 (35.5)
SDAI, mean (SD)	11.7 (5.6)
CDAI, mean (SD)	11.4 (5.0)
CRP < 5 mg/L, n (%)	48 (63.2)
CRP ≥ 5 mg/L, n (%)	28 (36.8)
CRP, mg/L, median (IQR)	3.3 (2.1–6.0)
Treatment	
Synthetic DMARDs, n (%)	45 (59.2)
Biological DMARDs, n (%)	56 (73.7)
Corticosteroids at cut-off, n (%)	44 (57.9)
Polypharmacy, n (%)	69 (90.8)

Abbreviations: RA: Rheumatoid arthritis; SD: standard deviation; Age-CCI: age-adjusted Charlson Comorbidity Index; DAS28-ESR: 28-joint Disease Activity Score with erythrocyte sedimentation rate; SDAI: Simple Disease Activity Index; CDAI: Clinical Disease Activity Index; CRP: C-reactive protein; DMARDs, disease-modifying antirheumatic drugs.

**Table 2:** Nutritional characteristics, physical function, and quality of life in 76 older patients (≥65 years) with RA.

Variable	RA ≥ 65 Years = 76
Nutritional status	
MNA, mean (SD)	12.3 (2.0)
MNA classification	
Normal, n (%)	52 (68.4)
Impaired nutritional status, n (%)	24 (31.5)
Risk of malnutrition, n (%)	22 (28.9)
Malnutrition, n (%)	2 (2.6)
GNRI, mean (SD)	93.5 (7.7)
GNRI classification	
Normal, n (%)	17 (22.4)
Low risk of malnutrition, n (%)	31 (40.8)
Moderate risk of malnutrition, n (%)	23 (30.3)
High risk of malnutrition, n (%)	5 (6.6)
Total proteins, g/L, mean (SD)	6.8 (0.5)
Albumin, g/L, mean (SD)	4.1 (0.4)
Albumin < 3.8 g/L, n (%)	7 (9.2)
Hemoglobin, mg/dL, median (IQR)	13.2 (12.2–14.1)
Calcium, mg/dL, mean (SD)	11.8 (13.8)
Vitamin D, ng/mL, mean (SD)	315.5 (258.2–404.2)
Vitamin B12, pg/mL, median (IQR)	30.8 (15.3)
BMI, kg/m2, mean (SD)	28.1 (5.0)
Obesity (BMI ≥ 30)	30 (39.5)
Quality of life	
EQ-5D-5L, median (IQR) (0–1)	0.53 (0.31–0.71)
EQ-VAS, median (IQR)	55.0 (41.2–69.0)
Physical function	
IPAQ, METs, median (IQR)	260.0 (0.0–630.0)
HAQ-DI, score 0–3, mean (SD)	1.282 (0.798)
Functional class	
Steinbrocker I, n (%)	22 (28.9)
Steinbrocker II, n (%)	38 (50.0)
Steinbrocker III, n (%)	15 (19.7)
Steinbrocker IV, n (%)	1 (1.3)
SPPB, median (IQR)	7.0 (5.5–9.0)

Abbreviations: RA: Rheumatoid arthritis; MNA: Mini Nutritional assessment; GNRI: Geriatric Nutritional Risk Index; BMI: body mass index; EQ-5D-5L: 5-dimension 5-level European Quality of Life questionnaire; VAS: visual analog scale; IPAQ: International Physical Activity Questionnaire; HAQ-DI: Health Assessment Questionnaire Disability Index; SPPB: Short Physical Performance Battery.

**Table 3:** Factors associated with malnutrition in 76 older patients (≥65 years) with RA.

Variable	RA with Normal Nutrition n = 52	RA with Impaired Nutritional Status * n = 24	*p*-Value
Epidemiological characteristics			
Female sex, n (%)	40 (76.9)	20 (83.3)	0.524
Age in years, mean (SD)	69.9 (3.6)	73.1 (6.2)	0.007
Age groups			0.003
65–69 yrs, n (%)	26 (50.0)	6 (25.0)	
70–79 yrs, n (%)	26 (50.0)	14 (58.3)	
80–90 yrs, n (%)	0 (0.0)	4 (16.7)	
Clinical characteristics			
Age-CCI, median (IQR)	3.0 (2.0–4.0)	3.0 (3.0–4.0)	0.062
Smoking			0.327
Non-smokers, n (%)	36 (69.2)	18 (75.0)	
Smokers, n (%)	3 (5.8)	3 (12.5)	
Former smokers, n (%)	13 (25.0)	3 (12.5)	
Body mass index, mean (SD)	29.5 (4.8)	27.5 (5.0)	0.111
Time since diagnosis of RA, years, mean (SD)	16.7 (7.2)	20.6 (8.3)	0.042
Positive rheumatoid factor (>10), n (%)	37 (71.2)	20 (83.3)	0.254
Positive anti-citrullinated peptide antibodies (>20), n (%)	38 (73.1)	17 (70.8)	0.839
Erosive disease, n (%)	44 (84.6)	21 (87.5)	0.740
Inflammatory disease activity			
DAS28-ESR at cut-off, score 0–10, mean (SD)	2.6 (1.0)	3.4 (1.0)	0.003
Remission or low activity, n (%)	37 (71.2)	12 (50.0)	
Moderate or high activity, n (%)	15 (28.8)	12 (50.0)	
SDAI, mean (SD)	10.6 (5.6)	14.1 (5.0)	0.010
CDAI, mean (SD)	10.4 (5.0)	13.8 (4.8)	0.013
CRP < 5 mg/L, n (%)	37 (71.2)	11 (45.8)	0.033
CRP ≥ 5 mg/L, n (%)	15 (28.8)	13 (54.2)	0.033
CRP, mg/L, median (IQR)	3.1 (2.3–5.6)	5.0 (1.7–6.8)	0.368
Treatment			
Synthetic DMARDs, n (%)	30 (57.7)	15 (62.5)	0.692
Biological DMARDs, n (%)	38 (73.1)	18 (75.0)	0.860
Corticosteroids at cut-off, n (%)	30 (57.7)	14 (58.3)	0.958
Polypharmacy, n (%)	46 (88.5)	23 (95.8)	0.302
Nutritional parameters			
GNRI, mean (SD)	94.8 (6.9)	90.0 (8.5)	0.012
GNRI classification			0.034
Normal, n (%)	15 (28.8)	2 (8.3)	
Low risk of malnutrition, n (%)	22 (42.3)	10 (41.7)	
Moderate risk of malnutrition, n (%)	15 (28.8)	8 (33.3)	
High risk of malnutrition, n (%)	1 (1.9)	4 (16.7)	
Albumin, g/L, mean (SD)	4.2 (0.3)	4.0 (0.4)	0.118
Albumin < 3.8 g/L, n (%)	2 (3.8)	5 (20.8)	0.017
Hemoglobin, mg/dL, median (IQR)	13.2 (12.4–14.1)	12.9 (11.9–13.6)	0.066
Calcium, mg/dL, mean (SD)	13.1 (10.8)	9.2 (0.5)	0.278
Vitamin D, ng/mL, mean (SD)	33.5 (16.1)	25.1 (11.7)	0.035
Vitamin B12, pg/mL, median (IQR)	322.0 (264.2–393.7)	293.0 (250.5–417.5)	0.558
Quality of life			
EQ-5D-5L, median (IQR) (0–1)	0.5 (0.4–0.7)	0.4 (0.1–0.5)	0.001
EQ-VAS, median (IQR)	60.0 (50.0–70.0)	50.0 (30.0–63.7)	0.063
Physical function			
IPAQ, METs, median (IQR)	360.0 (0.0–693.0)	0.0 (0.0–347.5)	0.014
HAQ-DI, score 0–3, mean (SD)	1.1 (0.7)	1.5 (0.8)	0.044
Functional class			0.093
Steinbrocker I, n (%)	19 (36.5)	3 (12.5)	
Steinbrocker II, n (%)	24 (46.2)	14 (58.3)	
Steinbrocker III, n (%)	9 (17.3)	6 (25.0)	
Steinbrocker IV, n (%)	0 (0.0)	1 (4.2)	
SPPB, median (IQR)	8.0 (6.0–9.0)	6.5 (4.7–7.2)	0.025

* At risk of malnutrition or malnourished. Abbreviations: RA: Rheumatoid arthritis; SD: standard deviation; Age-CCI: age-adjusted Charlson Comorbidity Index; DMARDs, disease-modifying antirheumatic drugs; GNRI: Geriatric Nutritional Risk Index; BMI: body mass index; DAS28-ESR: 28-joint Disease Activity Score with erythrocyte sedimentation rate; SDAI: Simple Disease Activity Index; CDI: Clinical Disease Activity Index; CRP: C-reactive protein; EQ-5D-5L: 5-dimension 5-level European Quality of Life questionnaire; VAS: visual analog scale; IPAQ: International Physical Activity Questionnaire; HAQ-DI: Health Assessment Questionnaire Disability Index; SPPB: Short Physical Performance Battery.

**Table 4:** Logistic regression model of factors associated with malnutrition in 76 older patients (≥65 years) with RA.

Variable	Univariate OR (95% CI)	Multivariate OR (95% CI)	*p*-Value
Age, years	1.088 (1.009, 1.172)	1.091 (1.006, 1.183)	0.035
Female sex	2.222 (0.788, 6.270)		
DAS28-ESR	2.108 (1.268, 3.506)	1.878 (1.086, 3.245)	0.024
EQ-VAS	0.181 (0.056, 0.591)	0.978 (0.957, 0.999)	0.041
IPAQ, METs	0.999 (0.999, 1.000)		
HAQ-DI	1.916 (1.004, 3.657)		
SPPB	0.839 (0.712, 0.989)		
GNRI	0.948 (0.899, 1.000)		

Naglekerke R^2^ = 0.205. Abbreviations: RA: Rheumatoid arthritis; DAS28-ESR: 28-joint Disease Activity Score with erythrocyte sedimentation rate; EQ-VAS: 5-dimension 5-level European Quality of Life questionnaire visual analog scale; IPAQ: International Physical Activity Questionnaire; HAQ-DI: Health Assessment Questionnaire Disability Index; SPPB: Short Physical Performance Battery; GNRI: Geriatric Nutritional Risk Index. Variables included in the equation: age, sex, DAS28-ESR, EQ-VAS, IPAQ, HAQ, and SPPB.

**Table 5:** Linear regression model of factors associated with malnutrition according to the MNA in 76 older patients (≥65 years) with RA.

Predictor	Univariate		Multivariate		
	B	95% CI	B	95% CI	*p*-Value
Age in years	−0.163	−0.251, −0.075	−0.121	−0.201, −0.041	0.004
Female sex	−0.471	−1.591, 0.650			
DAS28-ESR	−0.690	−1.083, −0.297	−0.427	−0.792, −0.062	0.023
EQ-VAS	0.033	0.019, 0.048	0.025	0.003, 0.046	0.026
IPAQ, METs	0.001	0.0, 0.002			
HAQ-DI	−0.847	−1.392, −0.302			
SPPB	0.266	0.027, 0.505			
GNRI	0.040	0.018, 0.072	0.065	0.014, 0.115	0.014

Naglekerke R^2^ = 0.334. Abbreviations: RA: Rheumatoid arthritis; MNA: Mini Nutritional Assessment; DAS28-ESR: 28-joint Disease Activity Score with erythrocyte sedimentation rate; EQ-VAS: 5-dimension 5-level European Quality of Life questionnaire visual analog scale; IPAQ: International Physical Activity Questionnaire; HAQ-DI: Health Assessment Questionnaire Disability Index; SPPB: Short Physical Performance Battery; GNRI: Geriatric Nutritional Risk Index. Variables included in the equation: age, sex, DAS28-ESR, EQ-VAS, IPAQ, HAQ, and SPPB.

## Data Availability

The datasets used and/or analyzed in the present study are available from the corresponding author upon reasonable request.

## References

[1] Zhang J.F., Ye X.L., Duan M., Zhou X.L., Yao Z.Z., Zhao J.X. (2020). Clinical characteristics of elderly and younger onset rheumatoid arthritis. Zhonghua Yi Xue Za Zhi.

[2] Mena-Vázquez N., Lisbona-Montañez J.M., Redondo-Rodriguez R., Mucientes A., Manrique-Arija S., Rioja J., Garcia-Studer A., Ortiz-Márquez F., Cano-García L., Fernández-Nebro A. (2022). Inflammatory profile of incident cases of late-onset compared with young-onset rheumatoid arthritis: A nested cohort study. Front. Med..

[3] Ruban T.N., Jacob B., Pope J.E., Keystone E.C., Bombardier C., Kuriya B. (2016). The influence of age at disease onset on disease activity and disability: Results from the Ontario Best Practices Research Initiative. Clin. Rheumatol..

[4] Tański W., Wójciga J., Jankowska-Polańska B. (2021). Association between Malnutrition and Quality of Life in Elderly Patients with Rheumatoid Arthritis. Nutrients.

[5] Engvall I.-L., Elkan A.-C., Tengstrand B., Cederholm T., Brismar K., Hafstrom I. (2008). Cachexia in rheumatoid arthritis is associated with inflammatory activity, physical disability, and low bioavailable insulin-like growth factor. Scand. J. Rheumatol..

[6] Metsios G.S., Stavropoulos-Kalinoglou A., Douglas K.M.J., Koutedakis Y., Nevill A.M., Panoulas V.F., Kita M., Kitas G.D. (2007). Blockade of tumour necrosis factor-alpha in rheumatoid arthritis: Effects on components of rheumatoid cachexia. Rheumatology.

[7] Manrique-Arija S., Mena-Vazquez N., Ureña I., Rioja J., Valdivielso P., Ginel-Mendoza L., Abad-Sánchez S., Jiménez-Núñez F.G., Oliver-Martos B., Fernandez-Nebro A. (2021). Cumulative inflammatory burden and obesity as determinants of insulin resistance in patients with established rheumatoid arthritis: Cross-sectional study. BMJ Open.

[8] Cano-García L., Manrique-Arija S., Domínguez-Quesada C., Vacas-Pérez J.C., Armenteros-Ortiz P.J., Ruiz-Vilchez D., Martín-Martín J.M., Redondo-Rodríguez R., García-Studer A., Ortiz-Márquez F. (2023). Sarcopenia and Nutrition in Elderly Rheumatoid Arthritis Patients: A Cross-Sectional Study to Determine Prevalence and Risk Factors. Nutrients.

[9] Aziz E.F., Javed F., Pratap B., Musat D., Nader A., Pulimi S., Alivar C.L., Herzog E., Kukin M.L. (2011). Malnutrition as assessed by nutritional risk index is associated with worse outcome in patients admitted with acute decompensated heart failure: An ACAP-HF data analysis. Heart Int..

[10] Yamaya M., Usami O., Nakayama S., Tode N., Yamada A., Ito S., Omata F., Momma H., Funakubo M., Ichinose M. (2020). Malnutrition, Airflow Limitation and Severe Emphysema are Risks for Exacerbation of Chronic Obstructive Pulmonary Disease in Japanese Subjects: A Retrospective Single-Center Study. Int. J. Chronic Obstr. Pulm. Dis..

[11] Polański J., Jankowska-Polańska B., Uchmanowicz I., Chabowski M., Janczak D., Mazur G., Rosińczuk J. (2017). Malnutrition and Quality of Life in Patients with Non-Small-Cell Lung Cancer. Adv. Exp. Med. Biol..

[12] García-Poma A., Segami M.I., Mora C.S., Ugarte M.F., Terrazas H.N., Rhor E.A., García E., Ramos M.P., Alva M., Castañeda I. (2007). Obesity is independently associated with impaired quality of life in patients with rheumatoid arthritis. Clin. Rheumatol..

[13] Fukuda W., Yamazaki T., Akaogi T., Hayashi H., Kusakabe T., Tsubouchi Y., Kawahito Y., Inoue M., Yoshikawa T. (2005). Malnutrition and disease progression in patients with rheumatoid arthritis. Mod. Rheumatol..

[14] Aletaha D., Neogi T., Silman A.J., Funovits J., Felson D.T., Bingham C.O., Birnbaum N.S., Burmester G.R., Bykerk V.P., Cohen M.D. (2010). 2010 Rheumatoid arthritis classification criteria: An American College of Rheumatology/European League Against Rheumatism collaborative initiative. Arthritis Rheum..

[15] Rubenstein L.Z., Harker J.O., Salvà A., Guigoz Y., Vellas B. (2001). Screening for undernutrition in geriatric practice: Developing the short-form mini-nutritional assessment (MNA-SF). J. Gerontol. A Biol. Sci. Med. Sci..

[16] De La Montana J., Miguez M. (2011). Suitability of the short-form Mini Nutritional Assessment in free-living elderly people in the northwest of Spain. J. Nutr. Health Aging.

[17] Bouillanne O., Morineau G., Dupont C., Coulombel I., Vincent J.-P., Nicolis I., Benazeth S., Cynober L., Aussel C. (2005). Geriatric Nutritional Risk Index: A new index for evaluating at-risk elderly medical patients. Am. J. Clin. Nutr..

[18] Gil-Bona J., Sabaté A., Miguelena Bovadilla J.M., Adroer R., Koo M., Jaurrieta E. (2010). Charlson index and the surgical risk scale in the analysis of surgical mortality. Cir. Esp..

[19] Charlson M.E., Pompei P., Ales K.L., MacKenzie C.R. (1987). A new method of classifying prognostic comorbidity in longitudinal studies: Development and validation. J. Chronic Dis..

[20] Mena-Vázquez N., Rojas-Gimenez M., Romero-Barco C.M., Gandía-Martínez M., Perez-Gómez N., Godoy-Navarrete F.J., Manrique-Arija S., Garcia-Studer A., Calvo-Gutiérrez J., Varela C.F. (2023). Analysis of comorbidity in rheumatoid arthritis-associated interstitial lung disease: A nested case-cohort study. Biomed. Pharmacother..

[21] Herdman M., Gudex C., Lloyd A., Janssen M., Kind P., Parkin D., Bonsel G., Badia X. (2011). Development and preliminary testing of the new five-level version of EQ-5D (EQ-5D-5L). Qual. Life Res..

[22] Rabin R., de Charro F. (2001). EQ-5D: A measure of health status from the EuroQol Group. Ann. Med..

[23] Tan X.L., Pugh G., Humby F., Morrissey D. (2019). Factors associated with physical activity engagement among adults with rheumatoid arthritis: A cross-sectional study. Musculoskelet. Care.

[24] Esteve-Vives J., Batlle-Gualda E., Reig A. (1993). Spanish version of the Health Assessment Questionnaire: Reliability, validity and transcultural equivalency. Grupo para la Adaptacion del HAQ a la Poblacion Espanola. J. Rheumatol..

[25] Steinbrocker O., Traeger C.H., Batterman R.C. (1949). Therapeutic criteria in rheumatoid arthritis. J. Am. Med. Assoc..

[26] Cruz-Jentoft A.J., Bahat G., Bauer J., Boirie Y., Bruyère O., Cederholm T., Cooper C., Landi F., Rolland Y., Sayer A.A. (2019). Sarcopenia: Revised European consensus on definition and diagnosis. Age Ageing.

[27] Prevoo M.L., van ’t Hof M.A., Kuper H.H., van Leeuwen M.A., van de Putte L.B., van Riel P.L. (1995). Modified disease activity scores that include twenty-eight-joint counts. Development and validation in a prospective longitudinal study of patients with rheumatoid arthritis. Arthritis Rheum..

[28] Lassale C., Batty G.D., Steptoe A., Cadar D., Akbaraly T.N., Kivimäki M., Kivimäki M., Zaninotto P. (2019). Association of 10-Year C-Reactive Protein Trajectories With Markers of Healthy Aging: Findings From the English Longitudinal Study of Aging. J. Gerontol. A Biol. Sci. Med. Sci..

[29] Wagner L., Haefeli W.E., Merle U., Lorenz H.-M., Hohmann N., Weiss J., Theile D. (2021). A nuclear factor kappa B reporter cell line used to evaluate ex vivo the net inflammatory effect of plasma samples from patients with rheumatoid arthritis, psoriasis, or COVID-19. Cytokine.

[30] Rasheed S., Woods R.T. (2014). An investigation into the association between nutritional status and quality of life in older people admitted to hospital. J. Hum. Nutr. Diet. Off. J. Br. Diet. Assoc..

[31] Olsen M.N., Tangvik R.J., Halse A.-K. (2020). Evaluation of Nutritional Status and Methods to Identify Nutritional Risk in Rheumatoid Arthritis and Spondyloarthritis. Nutrients.

[32] Elkan A.-C., Håkansson N., Frostegård J., Cederholm T., Hafström I. (2009). Rheumatoid cachexia is associated with dyslipidemia and low levels of atheroprotective natural antibodies against phosphorylcholine but not with dietary fat in patients with rheumatoid arthritis: A cross-sectional study. Arthritis Res. Ther..

[33] Elkan A.-C., Engvall I.-L., Cederholm T., Hafström I. (2009). Rheumatoid cachexia, central obesity and malnutrition in patients with low-active rheumatoid arthritis: Feasibility of anthropometry, Mini Nutritional Assessment and body composition techniques. Eur. J. Nutr..

[34] Agarwal E., Miller M., Yaxley A., Isenring E. (2013). Malnutrition in the elderly: A narrative review. Maturitas.

[35] Kaiser M.J., Bauer J.M., Ramsch C., Uter W., Guigoz Y., Cederholm T., Thomas D.R., Anthony P., Charlton K.E., Maggio M. (2009). Validation of the Mini Nutritional Assessment short-form (MNA-SF): A practical tool for identification of nutritional status. J. Nutr. Health Aging.

[36] Cuervo M., García A., Ansorena D., Sánchez-Villegas A., Martínez-González M., Astiasarán I., Martínez J. (2009). Nutritional assessment interpretation on 22,007 Spanish community-dwelling elders through the Mini Nutritional Assessment test. Public Health Nutr..

[37] Markaki A.G., Gkiouras K., Papakitsos C., Grammatikopoulou M.G., Papatsaraki A., Ioannou R., Tsagkari A., Papamitsou T., Bogdanos D.P. (2020). Disease Activity, Functional Ability and Nutritional Status in Patients with Rheumatoid Arthritis: An Observational Study in Greece. Mediterr. J. Rheumatol..

[38] Tokumoto H., Tominaga H., Arishima Y., Jokoji G., Akimoto M., Ohtsubo H., Taketomi E., Sunahara N., Nagano S., Ishidou Y. (2018). Association between Bone Mineral Density of Femoral Neck and Geriatric Nutritional Risk Index in Rheumatoid Arthritis Patients Treated with Biological Disease-Modifying Anti-Rheumatic Drugs. Nutrients.

[39] Bennett J.L., Pratt A.G., Dodds R., Sayer A.A., Isaacs J.D. (2023). Rheumatoid sarcopenia: Loss of skeletal muscle strength and mass in rheumatoid arthritis. Nat. Rev. Rheumatol..

[40] Fukuda W., Omoto A., Ohta T., Majima S., Kimura T., Tanaka T., Kohno M., Kawahito Y. (2013). Low body mass index is associated with impaired quality of life in patients with rheumatoid arthritis. Int. J. Rheum. Dis..

[41] Kremers H.M., Nicola P.J., Crowson C.S., Ballman K.V., Gabriel S.E. (2004). Prognostic importance of low body mass index in relation to cardiovascular mortality in rheumatoid arthritis. Arthritis Rheum..

[42] López-Gómez J.J., Calleja-Fernández A., Ballesteros-Pomar M.D., Vidal-Casariego A., Brea-Laranjo C., Fariza-Vicente E., Arias-García R.M., Cano-Rodríguez I. (2011). Screening of the nutritional risk in elderly hospitalized patients with different tools. Endocrinol. Nutr..

[43] Abd Aziz N.A.S., Mohd Fahmi Teng N.I., Kamarul Zaman M. (2019). Geriatric Nutrition Risk Index is comparable to the mini nutritional assessment for assessing nutritional status in elderly hospitalized patients. Clin. Nutr. ESPEN.

[44] Cereda E. (2012). Mini nutritional assessment. Curr. Opin. Clin. Nutr. Metab. Care.

